# Visual-textual integration in LLMs for medical diagnosis: A preliminary quantitative analysis

**DOI:** 10.1016/j.csbj.2024.12.019

**Published:** 2024-12-22

**Authors:** Reem Agbareia, Mahmud Omar, Shelly Soffer, Benjamin S. Glicksberg, Girish N. Nadkarni, Eyal Klang

**Affiliations:** a Ophthalmology Department, Hadassah Medical Center, Jerusalem, Israel; b Division of Data-Driven and Digital Medicine (D3M), Icahn School of Medicine at Mount Sinai, New York, NY, USA; c Institute of Hematology, Davidoff Cancer Center, Rabin Medical Center, Petah-Tikva, Israel

**Keywords:** Artificial intelligence, Medical diagnosis, Multimodal learning, Large language models, Visual data integration

## Abstract

**Background and aim:**

Visual data from images is essential for many medical diagnoses. This study evaluates the performance of multimodal Large Language Models (LLMs) in integrating textual and visual information for diagnostic purposes.

**Methods:**

We tested GPT-4o and Claude Sonnet 3.5 on 120 clinical vignettes with and without accompanying images. Each vignette included patient demographics, a chief concern, and relevant medical history. Vignettes were paired with either clinical or radiological images from two sources: 100 images from the OPENi database and 20 images from recent NEJM challenges, ensuring they were not in the LLMs' training sets. Three primary care physicians served as a human benchmark. We analyzed diagnostic accuracy and the models' explanations for a subset of cases.

**Results:**

LLMs outperformed physicians in text-only scenarios (GPT-4o: 70.8 %, Claude Sonnet 3.5: 59.5 %, Physicians: 39.5 %, p < 0.001, Bonferroni-adjusted). With image integration, all improved, but physicians showed the largest gain (GPT-4o: 84.5 %, p < 0.001; Claude Sonnet 3.5: 67.3 %, p = 0.060; Physicians: 78.8 %, p < 0.001, all Bonferroni-adjusted). LLMs altered their explanatory reasoning in 45–60 % of cases when images were provided.

**Conclusion:**

Multimodal LLMs showed higher diagnostic accuracy than physicians in text-only scenarios, even in cases designed to require visual interpretation, suggesting that while images can enhance diagnostic accuracy, they may not be essential in every instance. Although adding images further improved LLM performance, the magnitude of this improvement was smaller than that observed in physicians. These findings suggest that enhanced visual data processing may be needed for LLMs to achieve the degree of image-related performance gains seen in human examiners.

## Graphical Abstract

**Figure:**
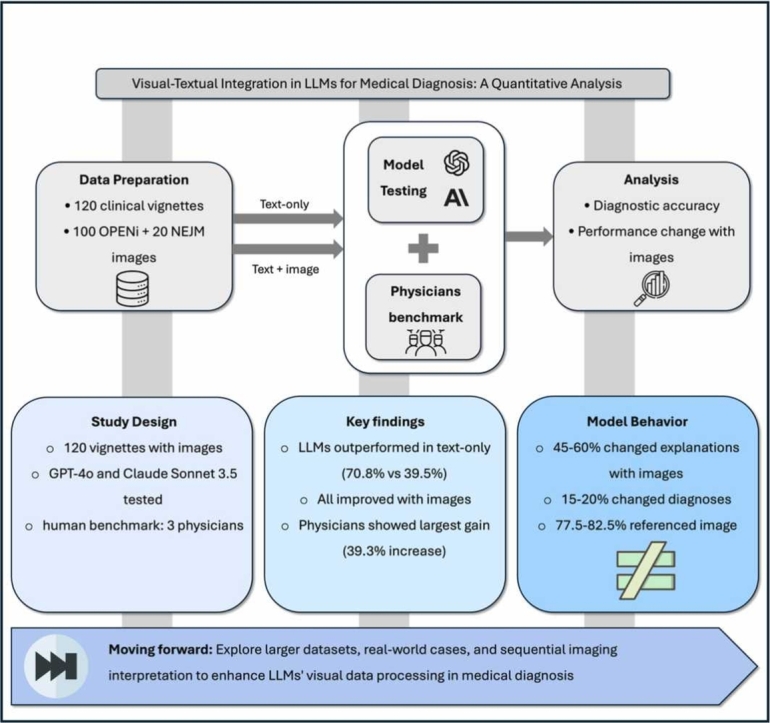

## 1 Introduction

Visual data, such as patient examinations or medical imaging, is central to clinical diagnosis [1]. Traditionally, clinicians combine textual information (e.g., medical history, symptoms) with visual cues (e.g., imaging findings) to form accurate diagnostic impressions. This integrated approach can often uncover subtleties that might not be evident from text alone [1].

Multimodal Large Language Models (LLMs) extend the capabilities of traditional language models by processing and integrating both textual and visual inputs [2], [3]. These models, often leveraging vision transformers and cross-attention mechanisms, map visual features onto a shared representation space that aligns with linguistic information [4]. For example, a multimodal LLM can analyze both a patient's written symptoms and an X-ray image, potentially enhancing diagnostic accuracy [5]. Early studies suggest that such models hold promise for automated image interpretation and differential diagnosis generation, yet their effectiveness and stability in real-world clinical settings remain uncertain.

A key question is whether LLMs prioritize textual information over visual cues [6]. Because they are generally pre-trained on large, text-dominated corpora, they may inherently rely more on textual patterns and context when making clinical judgments [7], [8]. Additionally, we suspect that the complexity of mapping rich visual information to a textual domain, combined with fewer standardized large-scale medical imaging datasets, might lead models to underutilize or misinterpret visual data [9].

While the integration of visual information into LLMs holds promise, its application in healthcare remains understudied. It is unclear how effectively these models incorporate visual clinical and imaging data alongside textual information when making medical diagnoses [10], [11]. Understanding how these models balance textual and visual inputs is crucial for refining their design and ensuring they contribute effectively to clinical decision-making.

This study evaluates the performance of multimodal LLMs in integrating textual and visual information for diagnostic purposes. By examining scenarios that require visual interpretation, we aim to determine whether these models can leverage images to improve diagnostic accuracy or whether they predominantly rely on textual cues. In doing so, we seek to clarify their current limitations and guide future improvements in multimodal model development.

## 2 Materials and methods

### 2.1 Study design and data preparation

**Fig. 1:**
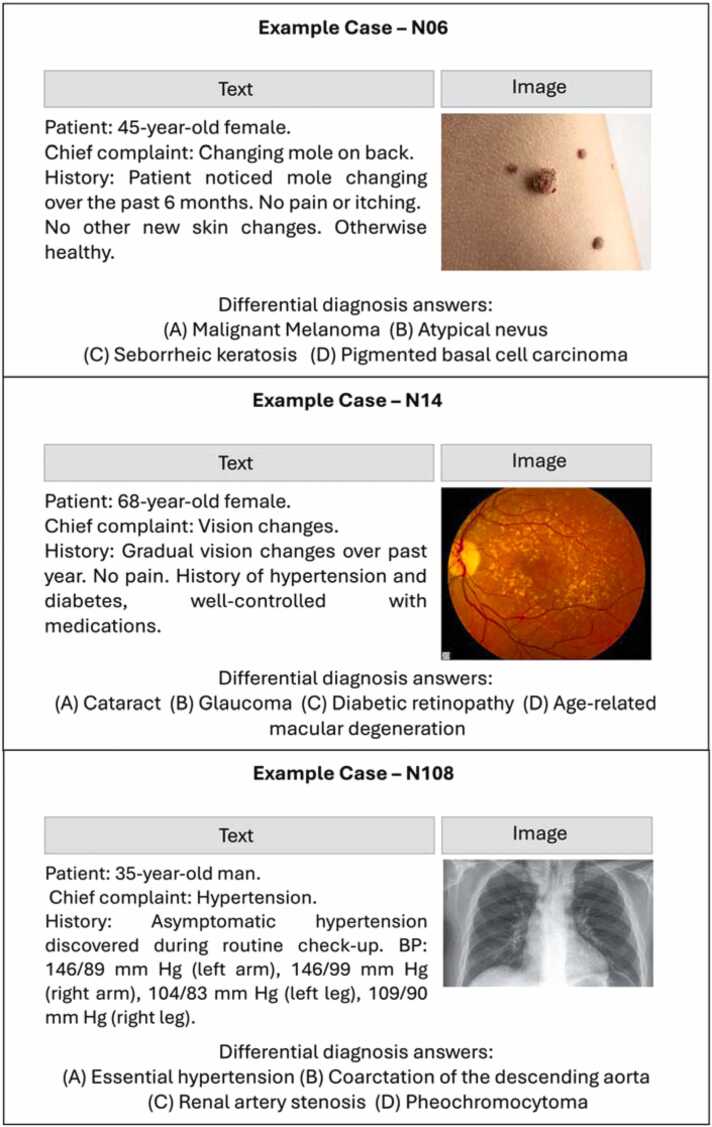
Example cases.

We evaluated LLMs' performance in diagnosing clinical cases using textual and visual data. Our dataset comprised 120 clinical vignettes, each with patient demographics, chief complaint, and relevant history. We included 100 images (80 clinical and 20 radiological images) from OPENi (https://openi.nlm.nih.gov). We also included 20 images from NEJM challenges published after March 2024, ensuring that they were not part of the LLMs' training data, as the cutoff knowledge of the included models was December 2023 (Fig. 1 presents an example case).

wo board-certified physicians chose images and wrote corresponding clinical vignettes, ensuring coverage of various medical fields and imaging modalities. Each vignette included demographics, a chief concern, and relevant history. Four differential diagnoses (one correct and three plausible alternatives) were then assigned to each case. We avoided including overt diagnostic clues in the text, reflecting scenarios where imaging is important. Two doctors wrote and cross-validated each case, following epidemiological guides for chief concerns and presenting symptoms [12]. We adhered to a systematic and consistent guidelines for creating each clinical vignettes [13], and used GPT-4 API for proofreading and validation [14]. (A complete flowchart of creating the case vignettes can be found in the supplement).

## 3 Explanations stress test

For a subset of 40 cases, we asked the models to provide explanations for their diagnoses in both text-only and text+imaging scenarios. We defined a ‘changed explanation’ as one in which the model’s reasoning or details differed significantly after images were introduced, regardless of whether the final diagnosis changed. Two physicians reviewed these explanations to determine if the models explicitly referenced visual data and thus genuinely integrated the images into their reasoning. This served as a stress-test to assess whether the models truly combined textual and visual inputs in their diagnostic process.

### 3.1 Model selection and implementation

We selected two multimodal LLMs for our study, accessed on August 2024: GPT-4o and Claude Sonnet 3.5. The implementation was carried out using Python 3.9, utilizing the OpenAI API (version 1.3.5) for GPT models and the Anthropic API (version 0.2.8) for Claude Sonnet 3.5. Data processing and numerical operations were performed using Pandas (version 1.5.3) and NumPy (version 1.23.5) libraries. Images were supplied as base64-encoded data appended to the prompt. No additional image preprocessing or augmentation was performed.

By comparing model performance between text-only and text image presentations, we aimed to isolate the contribution of the visual data. Since the diagnoses in our validated dataset was deemed often to rely on imaging, this comparison allowed us to determine whether adding the image substantially improved the models’ diagnostic accuracy beyond what could be inferred from text alone. We used a standardized prompt across all models, found in the **Supplement**.

### 3.2 Human benchmark

To benchmark against human performance, three board-certified primary care physicians independently evaluated all cases in both the text-only and the text + imaging formats. Their responses were collected using a custom-built web interface to ensure consistency in presentation.

### 3.3 Data analysis

We calculated descriptive statistics, including the number of correct answers for each model and for physicians, separately for text-only and text+imaging inputs. Before conducting statistical tests, we checked for normality and found no violation that would invalidate the use of parametric methods. We then performed paired t-tests to compare performance between text-only and text+imaging conditions for each model and for physicians. To compare the magnitude of performance improvement across all models and physicians, we conducted a one-way ANOVA followed by post-hoc pairwise t-tests with Bonferroni correction. All reported p-values reflect the Bonferroni-adjusted values. Corrected p-values below 0.005 were considered statistically significant.

The R packages used for analysis included tidyverse (version 1.3.2) and stats (version 4.2.2).

## 4 Results

### 4.1 Overview of the database

The 100 clinical cases from the OPENi database included 62 clinical images, primarily depicting various skin findings (46 cases, e.g., actinic keratosis, seborrheic dermatitis, Lyme disease rash), as well as a small number of ECGs [4], ocular findings (e.g., conjunctivitis, chalazion), and endoscopic images (6 cases, such as ulcerative colitis or esophageal cancer). The remaining 32 images from this dataset were radiological studies, consisting of CT [6], MRI [4], ultrasound [11], and X-ray [11] images. In the validation subset of the NEJM cases, all 20 images were clinical photographs.

### 4.2 Model and physicians overall performance

**Fig. 2:**
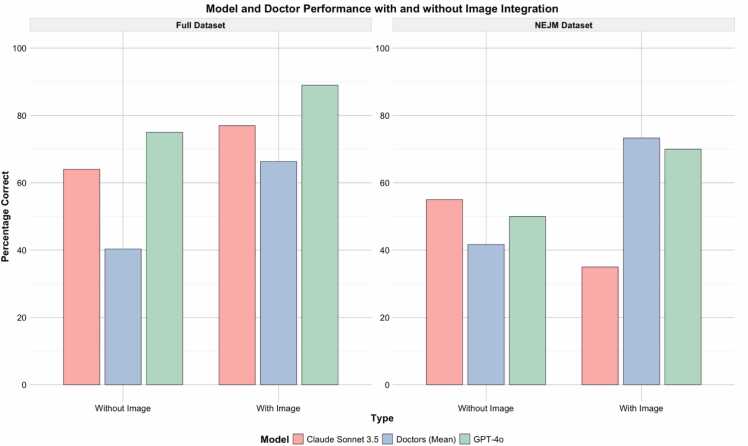
Performances across datasets, models and physicians.

Across the combined datasets, GPT-4o’s overall performance improved from 70.8 % ( ± 12.95 %) to 84.5 % ( ± 7.75 %) with image integration, a 13.7 % increase (p < 0.001). Claude Sonnet 3.5 improved from 59.5 % ( ± 12.95 %) to 67.3 % ( ± 7.75 %), a 7.8 % increase (p = 0.06). Physicians showed the largest improvement, from 39.5 % ( ± 12.95 %) to 78.8 % ( ± 7.75 %), a 39.3 % increase (p < 0.001). In the Full dataset, GPT-4o achieved 75 % ( ± 10.6 %) without images and 89 % ( ± 4.0 %) with images; Claude Sonnet 3.5 improved from 64 % ( ± 10.6 %) to 77 % ( ± 4.0 %), and physicians from 42.3 % ( ± 10.6 %) to 70.7 % ( ± 4.0 %) (Fig. 2).

### 4.3 Performance change with image integration

For GPT-4o, performance significantly improved with image integration. In the Full dataset, the correct response rate increased by 14 % (p < 0.001), and in the NEJM dataset, it increased by 20 % (p < 0.001). Claude Sonnet 3.5 showed a 13 % improvement in the Full dataset, though this change was not statistically significant (p = 0.060). However, Claude Sonnet 3.5's performance in the NEJM dataset dropped significantly with image integration, decreasing from 55 % correct in the text-alone condition to 35 % correct in the text + image condition (p < 0.001).

Across the combined datasets, GPT-4o's overall performance improved from 70.8 % to 84.5 %, while Claude Sonnet 3.5 improved from 59.5 % to 67.3 % (Table 1).

**Table 1:** Performance comparison of models and physicians in text alone vs. text + image conditions across datasets.

**Model**	**Dataset**	**Text Alone (Mean ± SD)**	**Text + Image (Mean ± SD)**	**Difference (%)**	**Adjusted p-value**
**GPT−4o**	Full	75.0 ± 10.6	89.0 ± 4.0	14.0	< 0.001
	NEJM	50.0 ± 15.3	70.0 ± 11.5	20.0	< 0.001
	Combined	70.8 ± 12.95	84.5 ± 7.75	13.7	< 0.001
**Claude Sonnet 3.5**	Full	64.0 ± 10.6	77.0 ± 4.0	13.0	0.06
	NEJM	55.0 ± 15.3	35.0 ± 11.5	−20	< 0.001
	Combined	59.5 ± 12.95	67.3 ± 7.75	7.8	0.06
**Physicians** **(Mean)**	Full	42.3 ± 10.6	70.7 ± 4.0	28.4	< 0.001
	NEJM	36.7 ± 15.3	71.7 ± 11.5	35.0	< 0.001
	Combined	39.5 ± 12.95	78.8 ± 7.75	39.3	< 0.001

### 4.4 Comparison of performance improvement between models and humans

The average improvement in performance for physicians with image integration was 28.3 % in the Full dataset and 35 % in the NEJM dataset. These improvements were statistically significant (p < 0.001).

In the combined datasets, physicians improved from 39.5 % to 78.8 %, a 39.3 % increase. The improvement for physicians was significantly greater than for GPT-4o in both datasets (p < 0.001), and significantly greater than for Claude Sonnet 3.5 in the NEJM dataset (p < 0.001). There was no significant difference in performance improvement between GPT-4o and Claude Sonnet 3.5 (p = 0.393).

### 4.5 Analysis of reasoning

In our analysis of model explanations, GPT-4o changed its explanations in 45 % of cases and its diagnosis in 20 % of cases when presented with images. Claude Sonnet 3.5 changed its explanations in 60 % of cases and diagnoses in 15 % of cases. Both models frequently referenced image evidence in their explanations, with GPT-4o doing so in 82.5 % of cases and Claude Sonnet 3.5 in 77.5 % of cases. The changes in explanations often involved shifts in reasoning, such as GPT-4o changing its diagnosis from malignant melanoma to seborrheic keratosis in a skin lesion case, or Claude Sonnet 3.5 altering its assessment from tension headaches to a brain tumor when presented with an MRI image (Table 2).

**Table 2:** Specific examples of changed explanations between textual only and textual with vision inputs.

**Model**	**Changed Explanations**	**Changed Diagnosis**	**Example Cases**
**GPT−4o**	18/40 (45 %)	8/40 (20 %)	• *Case 6 (Skin lesion):* • Text-only: Suggested malignant melanoma based on changing appearance and sun exposure history. • Text + imaging: Changed to seborrheic keratosis, describing a "raised, wart-like growth with a 'stuck-on' appearance".
			*Case 14 (Vision changes):* • Text-only: Suggested cataracts based on gradual vision changes and medical history. • Text + imaging: Changed to age-related macular degeneration, noting "the presence of drusen, which are yellow deposits under the retina".
**Sonnet 3.5**	24/40 (60 %)	6/40 (15 %)	*Case 13 (Abdominal discomfort):* • Text-only: Suggested irritable bowel syndrome based on intermittent symptoms and lack of alarming features. • Text + imaging: Changed to inflammatory bowel disease, noting "severe inflammation and ulcerations in the colon".
			*Case 18 (Headaches):* • Text-only: Suggested tension headaches based on the recurrent nature and lack of associated symptoms. • Text + imaging: Changed to brain tumor, stating "The MRI image shows a rounded, well-defined mass lesion in the brain".

## 5 Discussion

In this study, we evaluated the performance of multimodal LLMs when integrating textual and visual medical data. Across a broad range of clinical vignettes and imaging modalities, GPT-4o improved from about 71–85 % accuracy after images were provided, while Claude Sonnet 3.5 showed a more modest improvement from roughly 60–67 %. Physicians, starting at about 40 % accuracy in text-only conditions, improved significantly to nearly 79 % with image integration. Although LLMs performed better than physicians in text-only scenarios, their relative gain from including images was smaller. This suggests that while multimodal LLMs can incorporate visual cues, they do not yet extract as much value from images as human clinicians do.

These findings may highlight a key insight: current multimodal LLMs—despite their ability to parse and process both textual and visual inputs—may still rely heavily on text-based knowledge. This suggests LLMs possess extensive medical knowledge and potentially employ statistical approaches, choosing the most probable answer based on similar textual data in their training [15]. When images are added, their performance does improve, but not to the same extent as that of human physicians. This disparity suggests that the models’ visual reasoning capacity remains limited. This result, coupled with the observation that LLMs surpass physicians in text-only questions, implies that LLMs excel at recognizing textual patterns but have room to grow in leveraging visual information to mirror the diagnostic reasoning of skilled human observers. Physician's strengths in using visual data are based on their specific diagnostic techniques and experience, allowing them to interpret subtle, clinically relevant cues from images.

Our study adds to a growing body of literature showing that LLMs can reach or exceed physician-level performance in text-based assessments. For example, Katz et al. demonstrated that GPT-4 performed on par with or better than many trained physicians on standard board examinations [16]. Such findings illustrate that LLMs have matured enough in textual reasoning to merit serious consideration in clinical decision-support roles. However, prior evaluations have largely focused on text-only scenarios. Recent studies highlighting issues such as socio-demographic biases and hallucinations also primarily examine LLMs in text-based settings [17], [18], [19]. This context is crucial because integrating images introduces another layer of complexity—our study specifically probes this visual dimension. By moving beyond purely textual inputs, we provide insights into how LLMs handle multimodal data and whether known limitations from text-based tasks persist or evolve when visual information is involved. Our results suggest that while LLMs continue to demonstrate strong textual capabilities, their integration of imaging data is less robust. This underscores the need to broaden current evaluation frameworks to multimodal scenarios, ensuring that these models are tested and improved under conditions that more closely resemble real-world clinical environments.

Recent advances in multimodal LLMs that combine vision and language capabilities have been fueled by training on massive, web-scale image-text datasets [20], [21]. Models like LLaVA-Med, PMC-VQA, Med-Flamingo, and PeFoMed represent efforts to adapt generalist vision-language systems for medical applications [20], [21]. Research has shown that general-domain M-LLMs can learn robust internal representations of medical images, in some cases rivaling or surpassing specialized image classifiers [21]. Similarly, Agbareia et al. demonstrated that few-shot prompting can enhance OCT image interpretation, improving classification accuracy [22]. Our work complements these findings by focusing on how well these models integrate text and image data together for a single clinical decision, rather than just classifying isolated images. Our goal was unique in trying to quantify how much the models combine textual and visual information and precisely how their performance changes when images are introduced to scenarios designed to require them.

The diverse range of medical fields represented in our image dataset, contrasted with our primary care physician panel, presents both a limitation and an insight. While primary care physicians are trained across multiple specialties [23], [24], their performance might not fully represent specialist-level image interpretation. However, this scenario mirrors real-world primary care, where generalists encounter a wide range of conditions [23]. The LLMs' performance across these diverse cases showcases their potential as versatile diagnostic support tools across medical specialties.

LLMs outperforming physicians in question answering is well-documented. This aligns with existing research and benchmarks [16], [25], [26]. Some studies, particularly in radiology [27], suggest LLMs perform well in multimodal diagnosis [2], [27], yet we found no direct comparisons between multimodal, diverse diagnostic scenarios and text-only performance. Additionally, privacy concerns in medical imaging pose a significant challenge for LLM development. Training these models on large-scale human medical vision data while maintaining patient privacy is complex [28]. This limitation highlights the need for innovative approaches to data anonymization and synthetic data generation in medical AI research.

Our study has limitations. The sample size, particularly for the NEJM dataset is small, as it included only recently published cases to ensure that they were not part of the LLMs' training data. Additionally, artificially constructed cases- although validated by two physicians- may not fully represent real-world clinical complexity [13]. Further large-scale studies involving more diverse, real-world datasets are needed to better understand the true capabilities and boundaries of multimodal LLMs. The models’ susceptibility to biases and lack of contextual understanding also calls for continued refinement, with attention to transparent evaluation and mitigation strategies.

Moreover, our study focused only on static images and did not consider temporal sequences of images or longitudinal patient data. Many clinical decisions rely on tracking changes over time, so future research should explore how multimodal LLMs handle sequential imaging or integrate other modalities such as audio (e.g., heart or lung sounds) and video (e.g., gait analysis or endoscopic procedures) [29]. As multimodal technologies evolve, exploring their performance across additional data streams may unlock new diagnostic insights and further bridge the gap between human and AI-assisted clinical reasoning.

In conclusion, our results show that while multimodal LLMs can improve diagnostic accuracy by integrating images, their gains remain more modest than those achieved by human physicians. Future improvements in visual data processing, combined with larger and more representative datasets and the incorporation of additional modalities, will likely be necessary for LLMs to fulfill their potential as reliable, comprehensive tools in clinical decision-making aid.

## Author contributions

Reem Agbareia contributed to the conceptualization, methodology, and writing of the original draft. Mahmud Omar was involved in data analysis, interpretation, and review of the manuscript, and contributed equally to the research paper. Shelly Soffer provided expertise in clinical context, contributed to the discussion section, and reviewed the manuscript. Benjamin S. Glicksberg and Girish N. Nadkarni provided guidance on the integration of AI methods and contributed to the critical revision of the manuscript. Eyal Klang supervised the project, provided final approval of the manuscript, and ensured the integrity of the entire study. All authors have read and approved the final manuscript.

## Financial disclosure

This research received no specific grant from any funding agency in the public, commercial, or not-for-profit sectors.

## CRediT authorship contribution statement

**Reem Agbareia:** Writing – review & editing, Writing – original draft, Validation, Supervision, Software, Resources, Project administration, Methodology, Investigation, Formal analysis, Data curation, Conceptualization. **Shelly Soffer:** Writing – review & editing, Validation. **Mahmud Omar:** Writing – review & editing, Writing – original draft, Visualization, Validation, Supervision, Software, Resources, Project administration, Methodology, Investigation, Formal analysis, Data curation, Conceptualization. **Girish N Nadkarni:** Writing – review & editing, Validation, Supervision. **Benjamin S Glicksberg:** Writing – review & editing, Validation, Supervision. **Eyal Klang:** Writing – review & editing, Validation, Supervision, Investigation.

## Declaration of Competing Interest

The authors declare no conflicts of interest related to this study.
